# Case report: Supraglottic SCC with sphenoid and cavernous sinus metastases

**DOI:** 10.1016/j.ijscr.2024.110358

**Published:** 2024-09-26

**Authors:** N.A. O'Keeffe, E. Chiriac, A. Dias, A. Nae

**Affiliations:** a Department of Otolaryngology/Head & Neck Surgery, South Infirmary-Victoria University Hospital, Cork, Ireland; b Department of Radiology, South Infirmary-Victoria University Hospital, Cork, Ireland

**Keywords:** Sino-nasal cancer, Glottic cancer, Squamous cell carcinoma, Case report, Oncology, Metastases

## Abstract

**Introduction & importance:**

Primary Sino-nasal metastases are rare. The most common anatomical sites that metastasise to this region are the kidneys followed by the lungs, breast, thyroid and prostate. Metastases from laryngeal cancer are even rarer. We report a unique case of sphenoid and cavernous sinus metastases in a patient with glottic cancer. Herein we describe to the authors’ knowledge the first reported case of supraglottic metastases to the sphenoid and cavernous sinus. This study will help further our understanding metastatic spread outside of those well described in literature.

**Case presentation:**

A 75-year-old with a right neck swelling and hoarseness, treated for glottic SCC and represented with cranial nerve involvement suspicious for sinonasal metastases.

**Clinical discussion:**

Metastases to the sinonasal cavity are rare representing 3 % of head and neck malignancies. The most common primary sites include breast, colon, thyroid and prostate. Metastases from the larynx are exceedingly rare.

**Conclusion:**

This case report illustrates a rare case of Sino-nasal metastases from a patient with glottic SCC, it highlights an alternative metastatic pathway which often proves fatal.

## Highlights

• Primary Sino-nasal cancer is rare and has a high morbidity and mortality.
• Metastases to the sinuses are a very rare occurrence.
• This is the first reported case of glottic SCC to the sphenoid and cavernous sinus.
• Cavernous sinus involvement can result present with cranial nerve deficits.
• Management of Sino-nasal metastases is often palliative.

## 1 Introduction

Metastases to the sinuses and nasal cavity are exceedingly rare [1]. The most common types of primary cancers in the maxillary and ethmoid sinuses are adenocarcinoma and squamous cell carcinoma (SCC) [2]. Metastases typically occur in the maxillary sinus (33 %), followed by the sphenoid sinus (22 %), ethmoid (14 %), and frontal sinus (9 %). The most common cancerous sites that metastasize to this region are the kidney followed by the lung, breast, thyroid, and prostate. Patterns of laryngeal cancer spread are well reported, most often metastasizing to cervical lymph nodes with the most common site of distant metastasis to the lung followed by liver and bones [3]. To date there is very limited published literature describing sinonasal metastases from a laryngeal primary, Curry et al. reported described the scarcity of this presentation in 2001 with little reported on in the intervening period [4].

Metastases to the nasal cavity can either be single or multifocal. Symptoms may or may not be present, such as nasal obstruction, facial pain/pressure, headache, facial swelling, epistaxis, and cranial nerve deficits depending on the extent of invasion into local structures [1,5].

The objective of this case report was to highlight a unique case of sphenoid sinus metastases in a patient with glottic SCC which has not been previously reported on in the literature and help further our understanding of other routes of laryngeal squamous cell cancer metastases. This work has been reported in line with SCARE criteria [6].

## 2 Case presentation

**Fig. 1:**
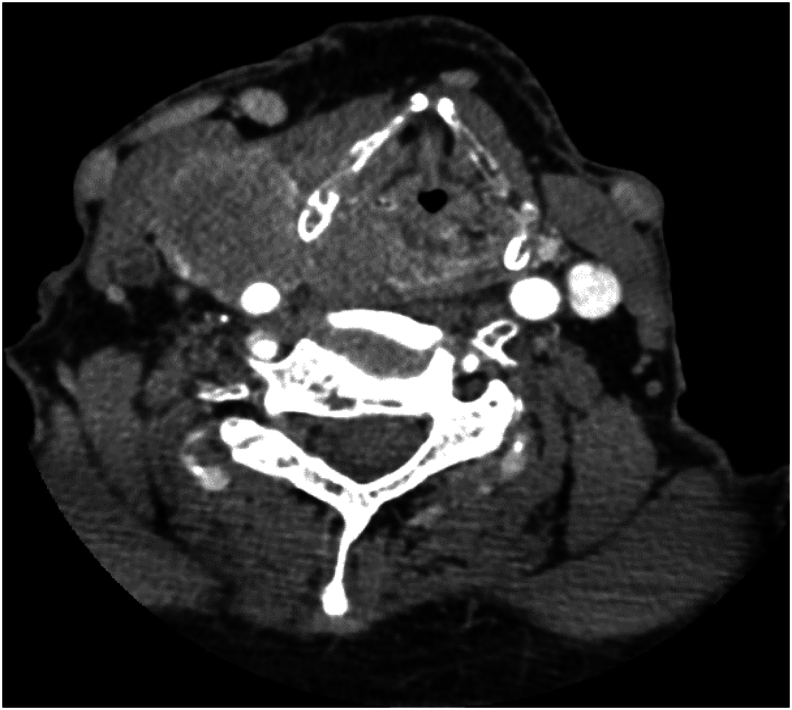
Axial CT neck with contrast showing large mucosal-bases enhancing lesion in the right hypopharynx involving the thyroid cartilage, with through-and-through local extension.

A 75-year-old Caucasian male presented with a right neck mass and hoarseness. He was a non-smoker, minimal alcohol intake with no significant past medical history and no regular medications. One week following presentation the patient underwent an ultrasound (US) and a fine needle aspiration and subsequently confirmed a malignant cytology. One week following confirmed pathology he underwent a panendoscopy which revealed a mass in the right aryepiglottic fold extending into the pyriform sinus and histology confirmed as SCC. Subsequent imaging in the form of computer tomography (CT) of his neck with contrast identified a 33 × 29 mm mucosal lesion of the right hypopharynx, involving the thyroid cartilage, with extra laryngeal extension and a 52 × 44 mm right level 3 adenopathy, involving the carotid bifurcation and internal jugular vein (as seen in Fig. 1).

**Fig. 2:**
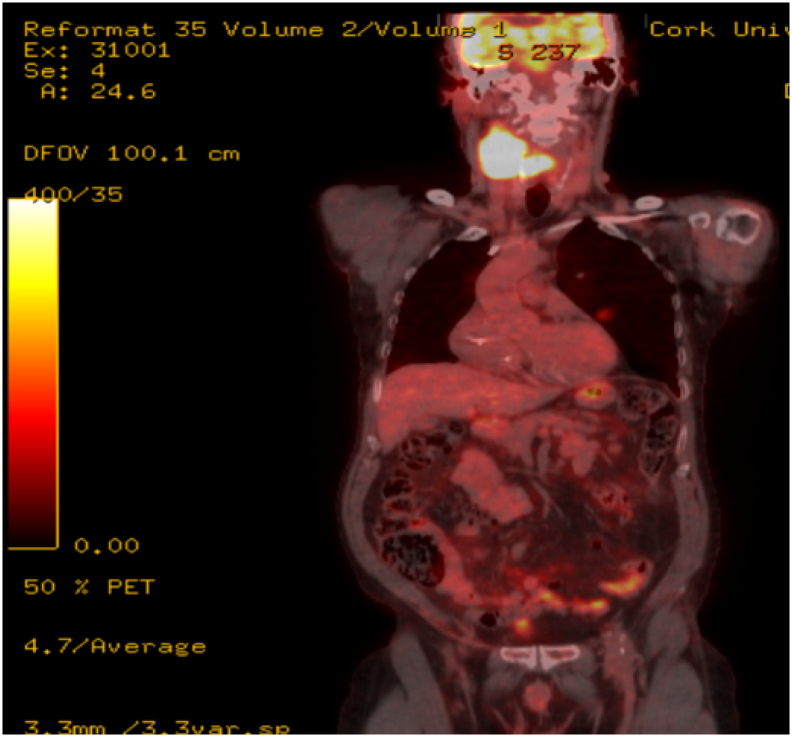
PET scan showing FDG avid uptake in the neck, liver deposits, and pulmonary nodules.

Three weeks following the initial diagnosis, a positron emission tomography (PET) scan confirmed the primary malignancy in the hypopharynx, the right level 3 necrotic adenopathy, and revealed the presence of pulmonary and liver metastasis. (Fig. 2). The patients staging, according to American Joint Committee on Cancer staging was T_4_N_2_M_1_ [7]. After multi-disciplinary team (MDT) discussion, he received high-dose palliative radiotherapy along with chemotherapy four weeks following initial diagnosis.

**Fig. 3:**
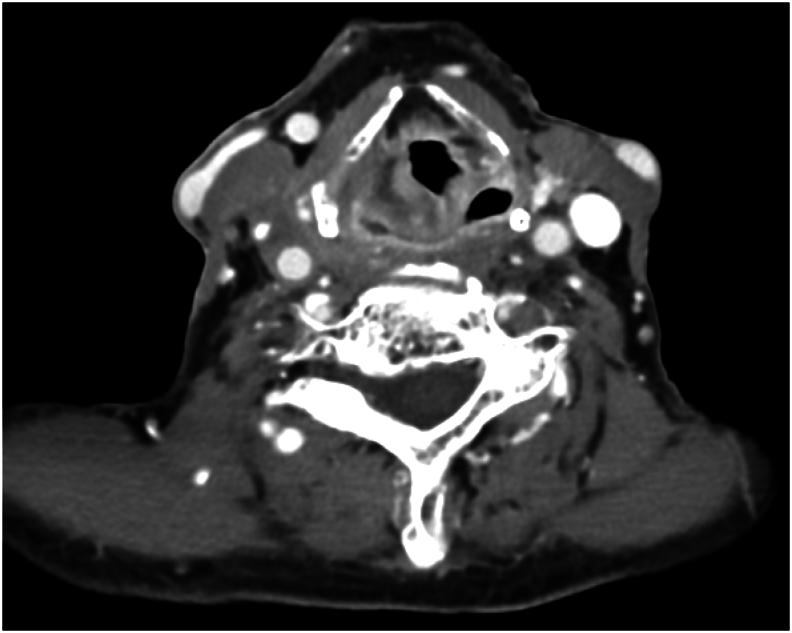
Axial CT showing improvement in primary lesion and neck adenopathy.

**Fig. 4:**
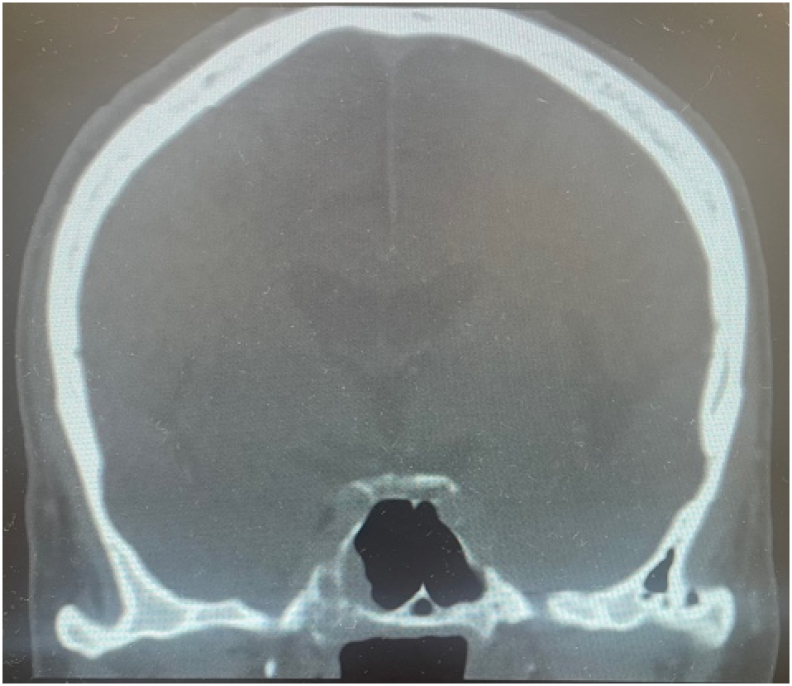
Coronal CT head showing 16 mm soft tissue lesion of right sphenoid with bony defects, extending into the cavernous sinus.

Two months after receiving radiotherapy, the follow up CT scan of his neck with contrast showed significant improvement of both primary lesion and right neck adenopathy (Fig. 3). Despite of this, 6 weeks post treatment the patient developed a complete right sided facial nerve palsy. A CT scan of his brain revealed a new, 16 mm soft tissue lesion in the sphenoid sinus, with bone destruction and minimal extension to the cavernous sinus area, indicating a possible secondary deposit (Fig. 4). Further endoscopic biopsy confirmed squamous cell carcinoma consistent with metastatic spread from laryngeal primary. His case was rediscussed at the multi-disciplinary team meeting, and he was commenced on palliative radiation therapy. The patient unfortunately passed away from widespread metastases 9 months following diagnosis.

## 3 Discussion

Malignancy of the sinonasal cavity is rare and only makes up about 3 % of head and neck malignancies. Most of these cases occur in the maxillary and ethmoid sinuses.

Furthermore, metastases to the sinonasal cavity are far less common than primary cancer in this location. If it does happen, the most likely source is renal cell carcinoma [8,9]. However, metastases from breast, colon, thyroid, and prostate cancer have also been documented [[10], [11], [12], [13]]. Laryngeal cancers have been reported to metastasise to the lung, ribs and lumbar spine most frequently [14].

Metastases to the cavernous sinus from head and neck cancer are rare, but have been reported in the literature [12,15]. Barret et al. reported the only case in literature of pyriform fossa SCC metastasis to sphenoid sinus. Barret et al. discuss possible routes of metastasis including hematogenous routes or through the lymphatics with subsequent distant dissemination and highlight the presence of such a lesion represents a high grade and advanced tumour [16]. We believe that this is the first case of laryngeal carcinoma with metastases to the sphenoid sinus and subsequent invasion of the cavernous sinus.

In general most cases of metastatic disease involve a single paranasal sinus, presentation that appears similar to primary sinonasal tumours [17]. The signs and symptoms of this can be non-specific, which may include nasal obstruction, epistaxis, diplopia, facial pressure and pain, and cranial nerve deficits [18].

The goal of treatment is to relieve symptoms and enhance the patient's quality of life. In most cases, the focus is on palliative care because of the widespread metastasis or systemic disease. For those with single, operable metastases, surgery is the best option. Other treatments, such as chemotherapy, radiation, or immunotherapy, are used for tumours that cannot be removed. Stereotactic radiation can be used in some circumstances where radiation has been performed previously [16,19]. Unfortunately, the prognosis for those with metastatic paranasal sinus disease is generally unfavourable [19]. However, understanding the mechanism and implications of such rare occurrences is important in the context of advanced head and neck cancers.

The authors are aware of the inherent weakness of this study including its retrospective nature and dependence on the interpretation of hand written medical notes which may add some element of bias.

## 4 Conclusion

To our knowledge, our case is the only instance of supraglottic SCC metastasizing to the sphenoid and cavernous sinus. Despite undergoing palliative chemo-radiotherapy, these metastases evolved rapidly. SCC is a highly aggressive disease that can spread to uncommon locations. The case highlights how clinicians must maintain a low index of suspicion in head and neck patients who present with unusual signs and symptoms unrelated to their site of primary cancer. Understanding this can lead to timely investigation, diagnosis, and treatment whether it be palliative or otherwise.

## Consent

Written informed consent was obtained from the patient's family for publication of this case report and accompanying images. A copy of the written consent is available for review by the Editor-in-Chief of this journal on request.

## Ethical approval

Study is exempt from ethical approval. Cork Regional Ethics Committee.

## Funding

This research did not receive any specific grant from funding agencies in the public, commercial, or not for profit sectors.

## Author contribution

N O'Keeffe – Original draft, writing, reviewing, and editing. E Chiriac - Editing of radiology input with image selection and captions. A Dias - Study concept and paper write up. A Nae - Study concept and paper write up.

## Guarantor

Mr. Andrew Dias, Consultant Otolaryngologist/Head & Neck Surgeon.

## Declaration of competing interest

No conflict of interest declared by authors.

## References

[1] Guntinas-Lichius O., Kreppel M., Stuetzer H., Semrau R., Eckel H., Mueller R. (2007). Single modality and multimodality treatment of nasal and paranasal sinuses cancer: a single institution experience of 229 patients. Eur. J. Surg. Oncol.(EJSO)..

[2] Llorente J.L., López F., Suárez C., Hermsen M.A. (2014). Sinonasal carcinoma: clinical, pathological, genetic and therapeutic advances. Nat. Rev. Clin. Oncol..

[3] Koroulakis A, Agarwal M. Laryngeal Cancer.[Updated 2021 Aug 11]. StatPearls [Internet] Treasure Island (FL): StatPearls Publishing. 2021.

[4] Curry M.P., Newlon J.L., Watson D.W. (2001). Cavernous sinus metastasis from laryngeal squamous cell carcinoma. Otolaryngol. Head Neck Surg..

[5] Prescher A., Brors D. (2001). Metastases to the paranasal sinuses: case report and review of the literature. Laryngo-rhino-otologie..

[6] Sohrabi C., Mathew G., Maria N., Kerwan A., Franchi T., Agha R.A. (2023). The SCARE 2023 guideline: updating consensus surgical CAse REport (SCARE) guidelines. Int. J. Surg..

[7] Zanoni D.K., Patel S.G., Shah J.P. (2019). Changes in the 8th edition of the American joint committee on Cancer (AJCC) staging of head and neck cancer: rationale and implications. Curr. Oncol. Rep..

[8] López F., Devaney K.O., Hanna E.Y., Rinaldo A., Ferlito A. (2016). Metastases to nasal cavity and paranasal sinuses. Head Neck.

[9] Sountoulides P., Metaxa L., Cindolo L. (2011). Atypical presentations and rare metastatic sites of renal cell carcinoma: a review of case reports. J. Med. Case Rep..

[10] Bernstein J.M., Montgomer W.W., Balogh K. (1966). Metastatic tumors to the maxilla, nose, and paranasal sinuses. Laryngoscope.

[11] Nahum A.M., Bailey B.J. (1963). Malignant tumors metastatic to the paranasal sinuses: case report and review of the literature. Laryngoscope.

[12] Madan R., Goyal S., Dinda A.K., Maohanti B.K. (2013). Papillary carcinoma of thyroid with paranasal sinus metastases. Clin. Cancer Investig J..

[13] Altiner H.I., Keskin H., Günel C. (2023).

[14] Alberti P., Bryce D. (1976).

[15] Bree R.D., Mehta D.M., Snow G.B., Quak J.J. (2001). Intracranial metastases in patients with squamous cell carcinoma of the head and neck. Otolaryngol. Head Neck Surg..

[16] Barrett G, Espeso A, Hickey S. Sphenoid sinus metastatic lesion from a pyriform fossa squamous cell carcinoma. J. Surg. Case Rep. 2011;2011(6):2-. 10.1093/jscr/2011.6.2 PMC3649251 24949697

[17] Weber A.L., Stanton A.C. (1984). Malignant tumors of the paranasal sinuses: radiologic, clinical, and histopathologic evaluation of 200 cases. Head Neck Surg..

[18] Matsumoto Y., Yanagihara N. (1982). Renal clear cell carcinoma metastatic to the nose and paranasal sinuses. Laryngoscope.

[19] Kamiński B., Kobiorska-Nowak J., Bień S. (2008). Distant metastases to nasal cavities and paranasal sinuses, from the organs outside the head and neck. Polish J. Otolaryngol..

